# Bioactivity and Biotechnological Overview of Naturally Occurring Compounds from the Dinoflagellate Family Symbiodiniaceae: A Systematic Review

**DOI:** 10.1155/2021/1983589

**Published:** 2021-12-17

**Authors:** Jeysson Sánchez-Suárez, Mariana Garnica-Agudelo, Luisa Villamil, Luis Díaz, Ericsson Coy-Barrera

**Affiliations:** ^1^Bioprospecting Research Group, School of Engineering, Universidad de La Sabana, Chía, Colombia; ^2^Bioorganic Chemistry Laboratory, Universidad Militar Nueva Granada, Cajicá, Colombia; ^3^Doctoral Program in Biosciences, School of Engineering, Universidad de La Sabana, Chía, Colombia

## Abstract

Marine invertebrates are a significant source of biologically active compounds. Recent studies have highlighted the role of microbiota associated with marine invertebrates in the production of bioactive compounds. Corals and sponges are the main marine invertebrates producing bioactive substances, and Symbiodiniaceae dinoflagellates are well-recognized endosymbionts with corals and sponges playing vital functions. The biological properties of Symbiodiniaceae-derived compounds have garnered attention in the past decades owing to their ecological implications and potentiality for bioprospecting initiatives. This study aims to systematically review studies on bioactivities and potential biotechnological applications of Symbiodiniaceae-derived compounds. The PRISMA guidelines were followed. Our study showed that anti-inflammatory and vasoconstrictive activities of Symbiodiniaceae-derived compounds have been the most investigated. However, very few studies have been published, with in vitro culturing of Symbiodiniaceae being the most significant challenge. Therefore, we surveyed for the metabolites reported so far, analyzed their chemodiversity, and discussed approaches to overcome culturing-related limitations.

## 1. Introduction

Although marine invertebrates have significant bioprospecting potential, it is associated with several methodological, ecological, and logistical challenges owing to limited sampling and the risk of changes in population dynamics [1]. These challenges are owing to the prolonged time and poor reproducibility of the complex environmental conditions required for the cultivation of invertebrate biomass (e.g., sponges) [2, 3]. Consequently, although marine invertebrates produce bioactive compounds of biotechnological utility, their biological characteristics limit large-scale harvesting of secondary metabolites in vitro [4]. In addition, challenges associated with large-scale production of the target bioactive compounds from a marine organism, that is, low yield on animal extraction and high costs and practical limitations of chemical synthesis, further limit the use of these compounds [5]. The origin of marine natural products (MNP) on bioprospecting studies on sessile or nonsessile marine invertebrates remains uncertain, which could be the organisms themselves, the associated microbiota, or the interaction between them [6]. The specialized metabolism of the associated microbiota remains to be studied.

Marine invertebrates are hosts to a rich and dynamic microbiota [7, 8] that are closely linked to the metabolism and survival of the invertebrates [9]. Endosymbiotic relationships result in mechanisms that promote mutual nutrition and predator defense; for example, sponges and their symbionts can produce toxic compounds to prevent attacks by other marine organisms [10, 11]. Studies that have reported on the production of such active compounds by the symbiotic microbiota also highlighted the need for further research [6, 12–14]. For instance, the endosymbiotic Symbiodiniaceae dinoflagellates growing on *Pseudopterogorgia elisabethae* produce pseudopterosins that provide the host with oxidative stress tolerance [15]. In addition, unknown free-living marine bacteria, frequently associated with the tunicate *Ecteinascidia turbinata*, express groups of essential genes for the synthesis of bioactive metabolites (e.g., trabectedin) that are under research as potential oncologic treatment agents [16].

The dinoflagellate family Symbiodiniaceae (phylum Myzozoa, class Dinophyceae) is a symbiotic organism under active research [17]. These endosymbiotic microalgae provide hosts with nutrients through photosynthesis and receive protection and inorganic compounds [18–20]. Their ecology focuses on host compatibility, which depends on the type of the associated host, their distribution, and variations in abiotic factors such as irradiance, depth, pH, and temperature [21]. The systematics of the family Symbiodiniaceae (formerly, the *Symbiodinium* genus) were recently revised, with new genera replacing the earlier clades. Currently, the *Symbiodinium* genus only includes clade A (considered a living fossil) [22]. This taxon is known to produce secondary metabolites involving compounds with unique chemical structures and activities. Although several genera are known, several remain undiscovered [23]. The association between extensive genetic diversity and metabolic processes also remains unclear [24].

Although the importance of associated microbiota, particularly of Symbiodiniaceae endosymbionts, in marine invertebrate metabolism is understood, most literature reviews have focused on the biology of these dinoflagellates or their ecological implications. Two reviews (Gordon and Leggat [25] and Kita et al. [26]) highlighted some bioactive metabolites isolated from some Symbiodiniaceae species. However, to our knowledge, this review is the first to systematically collect, summarize, and analyze the data available in the literature regarding Symbiodiniaceae-derived bioactive metabolites. This review focuses mainly on the chemodiversity of the reported naturally occurring compounds and their potential biotechnological applicability.

## 2. Methods

### 2.1. Search and Eligibility Criteria

We conducted a systematic search for literature on naturally occurring compounds isolated from Symbiodiniaceae family members, their bioactivities, and biotechnological potential. Scopus, the Web of Science, and PubMed databases were used. The following search query was used: ((symbiodinium OR zooxanthellae) AND (metabolite OR compound OR agent OR substance OR molecule) AND (activity OR potential OR bioactivity OR bioactive OR effect OR extract OR isolated OR isolate OR derived OR isolation OR biotechnology)). Inclusion criteria regardless of the year of publication were (a) high-quality original articles, (b) describing the isolation of secondary metabolites from cultures of microalgae of the Symbiodiniaceae family (former genus *Symbiodinium*), and (c) written in English.

### 2.2. Study Selection and Data Collection

Duplicate articles were removed, and the title and abstract of each study were independently filtered according to the eligibility criteria by 2 of the authors using the Rayyan QCRI tool, with the classification categorized as included, excluded, or maybe [27]. Papers matching the classification criteria were studied. Articles classified as “maybe” were finally classified after discussion and consensus. After initial classification, the full texts of the articles were analyzed, and the information was captured using a form to ensure comprehensive data collection and bias elimination.

### 2.3. Analysis of Retrieved Compounds

All compounds identified in the systematic review were represented using the simplified molecular-input-line-entry system (SMILES) notation, and these were enlisted to obtain a custom-made library. The SMILES-annotated compounds were incorporated in the software Osiris DataWarrior (Idorsia Pharmaceuticals Ltd., Switzerland, version 5.2.1) [28], which was used to determine the physicochemical properties: molecular weight (MW), octanol/water partition coefficient (cLogP), aqueous solubility (cLogS), hydrogen bond acceptor (*H*-acceptor), hydrogen bond donors (*H*-donors), total surface area (TSA), polar surface area (PSA), relative polar surface area (rPSA), and druglikeness.

## 3. Results and Discussion

### 3.1. General Findings

**Figure 1:**
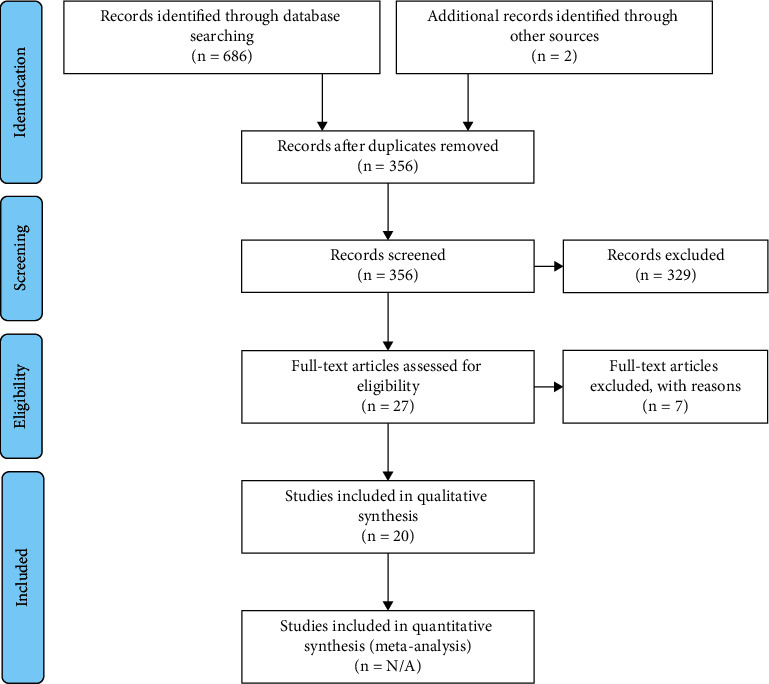
PRISMA flow diagram. Modified from Moher et al. [29]. Compliance with the items in the statement guideline is summarized in Table S1 in Supplementary Materials.

The literature search identified 686 articles; 160 of the retrieved articles were identified as duplicates among the 3 databases, leaving 356 unique articles. Titles and abstracts of these 356 articles were screened and filtered according to eligibility and selection criteria. After screening, only 27 studies were selected for data extraction. Based on full-text assessment and data extraction, 20 articles were finally included in this systematic review (Figure 1).

**Figure 2:**
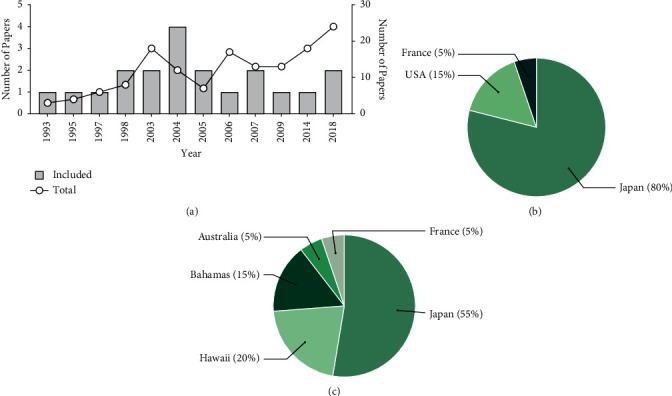
The number of studies published on naturally occurring compounds isolated from Symbiodiniaceae. (a) The distribution of published studies included (left axis) and screened (right axis) in this systematic review per year, (b) the number of publications according to corresponding authors' country, and (c) the number of publications according to the country of sample origin.

The included articles (*n* = 20) were published between 1993 and 2018 (Figure 2(a)). Most studies were published by authors in Japan (*n* = 16), followed by those in the USA and France (Figure 2(b)), which involved Symbiodiniaceae strains from 5 different countries (Figure 2(c)). To assess the publication behavior of bioprospecting studies on Symbiodiniaceae dinoflagellates, we compared all papers (that were screened) published within the same years. The number of studies increased during the same period, except for 2004 and 2005 (Figure 2(a)). However, a considerably low number of studies investigating Symbiodiniaceae-derived compounds and their bioactivity potential were published, with the highest of 4 studies published in 2004. This low academic productivity is associated with the small number of countries researching on this topic (Figure 2(b)), which is in contrast with the worldwide distribution of the Symbiodiniaceae species [30] and the metabolite diversity that marine invertebrates represent (e.g., corals and sponges) [1, 31–36], including their associated microbiota [12]. Japan had the highest contribution to research on this topic, with 45% of the included papers from Nagoya University. Focus on bioprospecting was minimal, with most studies focusing on topics such as ecology.

With regard to the diversity of Symbiodiniaceae isolates, 12 *Symbiodinium* strains have been studied for the specialized metabolism-derived products from these dinoflagellates (Table 1). The most reported strain was *Symbiodinium* sp Y-6 (isolated from the flatworm *Amphiscolops* sp), from which 7 compounds were reported (i.e., **1**, **2**, **6**, **8**, **9**, **21**, and **22**; see Table 2). Although Symbiodiniaceae dinoflagellates have been reported to be symbionts for various marine invertebrates [37], their symbiosis with cnidarians has been most investigated. Of interest, we found few studies (15.79%) on symbiosis with corals (i.e., *Sarcophyton glaucum*, *Pseudopterogorgia elisabethae,* and *Eunicea fusca*; see Table 1), which implies a subexploration of the Symbiodiniaceae family diversity.

### 3.2. Biologically Active Compounds

Among the 23 compounds identified from the selected publications (see chemical structures in Figures S1 and S2 in Supplementary Materials), biological activity evaluations were available only for 9 compounds (Table 2). We examined the vasoconstrictive and anti-inflammatory potency of these compounds. Vasoconstriction was the first bioactivity reported for Symbiodiniaceae-derived metabolites (i.e., **1** and **2**), with activity at concentrations >0.70 *μ*M [47]. Furthermore, metabolite **1** was also shown to induce platelet aggregation [55]. Metabolite **2** effects vasoconstriction through voltage-sensitive Ca^2+^ channels [46]. This finding is consistent with the calcium-dependent induction of platelet aggregation by metabolite **1** [56]. Compound **5** reportedly exhibits 3-fold higher vasoconstriction potency than compound **1** although its mechanism of action remains unclear [41]. To our knowledge, these compounds are yet being studied.

Metabolites **7**, **10**, **19**, and **23** have been reported to have anti-inflammatory potential. Metabolites **7** and **8** showed 65% and 32% cyclooxygenase-2 inhibition at 2 and 10 *μ*M concentrations, respectively [52, 53]. Compound **23** was evaluated basis its ability to suppress delayed-type hypersensitivity in mice [44]. Metabolite **10** has been reported to inhibit L-phosphatidylserine-stimulated protein kinase C activity [54]. Limited data availability makes estimating the biotechnological potential of these compounds (except for metabolite **23** [peridinin]) difficult.

Cytotoxicity of Symbiodiniaceae-derived metabolites has been studied. Two compounds, **6** and **9**, have been reported to show antineoplastic potential against carcinomas [42, 43]. Both compounds were evaluated on A432 and Nakata cell lines and exhibited inhibitory activity at concentrations <10 *μ*M. Compound **9** exhibited 16-fold higher potency [43]. Despite these encouraging results, we could not find further studies on the anticancer potential of these compounds.

The biological activities of compounds **3**, **4**, **8**, **11**–**18**, and **20**–**22** were not evaluated in the included papers; however, the bioactivity potential of some of these have been reported as they were isolated from other sources. In this regard, the phenolic diterpenes pseudopterosins (i.e., **14**–**17**) have been shown to have potential anti-inflammatory, analgesic [57], neuroprotective [58], and antioxidant [15, 59] activities. Similarly, compound **18** has shown anti-inflammatory properties superior to those of indomethacin [60]. Fuscol (**18**) has also been reported to exhibit moderate inhibition activity on elastase release assays [61]. These findings present an opportunity in the bioprospecting potential of Symbiodiniaceae dinoflagellates.

### 3.3. Chemodiversity of the Symbiodiniaceae-Derived Compounds

**Figure 3:**
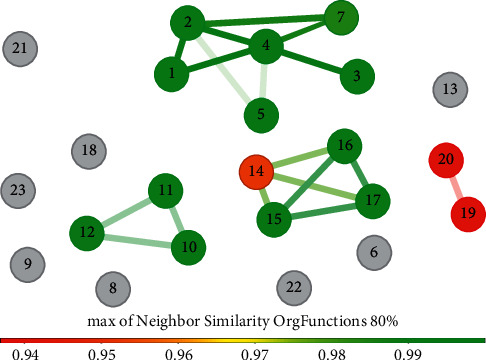
Similarity analysis of the Symbiodiniaceae-derived compounds. The similarity analysis was performed using the *OrgFunctions* descriptor. The color scale bar indicates the level of similarity from red (0.9) to green (1.0).

We clustered the retrieved compounds by structural similarity to analyze chemodiversity. Four clusters were formed, including 15 compounds, whereas 8 compounds had unique structural fingerprints (Figure 3). Most of the compounds corresponded to amides (i.e., **1**–**8**) followed by polyketides (i.e., **9**–**13**), diterpenes (i.e., **14**–**18**), and alkaloids (i.e., **19**–**22**); one was an apocarotenoid (i.e., **23**). These data are consistent with those from recent reports on the diversity and abundance of specialized metabolite biosynthesis genes in Symbiodiniaceae genomes, which show that polyketide synthase genes are the primary diversified gene clusters associated with specialized metabolism [62].

With regard to similarity analysis (Figure 3), compounds **3** and **4** in the larger group (i.e., **1**, **2**, **3**, **4**, **5**, and **7**) have been described as artifacts produced under chemically active conditions during the isolation process [41], leaving the number of natural compounds at 21. In addition, this group has compounds with vasoconstrictive bioactivity, possibly involving Ca^2+^ channels [46, 53, 55], reinforcing the role of the macrolactone moiety in this mechanism. With regard to the remaining compounds, except for **6**, clustering was anticipated by the metabolite type and trivial names (Table 2, Figure 3).

**Figure 4:**
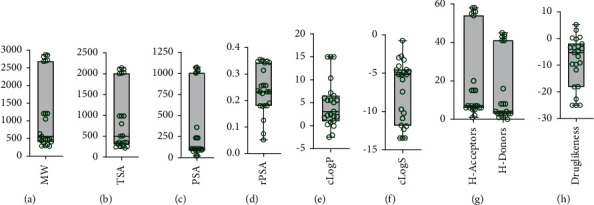
Structure-based physicochemical properties of the Symbiodiniaceae-derived compounds. Properties were estimated using the DataWarrior software. Box plots showing the distributions for (a) molecular weight (MW), (b) total surface area (TSA), (c) polar surface area (PSA), (d) relative polar surface area (rPSA), (e) octanol/water partition coefficient (cLogP), (f) aqueous solubility (cLogS), (g) hydrogen bond acceptor (H-acceptor) and hydrogen bond donors (H-donors), and (h) druglikeness.

Given the crucial role of physicochemical properties in the development of therapeutic agents [63], we examined the relevant properties of the selected compounds based on some descriptors (i.e., MW, cLogP, cLogS, *H*-acceptor, *H*-donors, and druglikeness; Figure 4). We identified 3 groups of compounds for MW, TSA, PSA, *H*-acceptors, and *H*-donors, whereas rPSA, cLogP, cLogS, and druglikeness showed a wider distribution. For instance, only 4 compounds had positive druglikeness values (i.e., **16**, **17**, **22**, and **23**), and the remaining compounds displayed values between −0.2 and −25.0 (Figure 4(h)). An implication of these results is the attrition associated with a high likelihood of failures in further research stages.

The compounds were classified as low (approximately 500 Da), medium (approximately 1200 Da), or large (approximately 2700 Da) MW compounds. Most compounds (61.90%, excluding **3** and **4**) were in the low MW group, which is desirable in drug development. However, several compounds in this group remain unevaluated for bioactivity potential (i.e., **8**, **13**, **20**, **21**, and **22**). These compounds, except **22**, were isolated in quantities <5 mg (Table 3), which could have limited their biological screening. In contrast, the compounds from dinoflagellates have been recognized to have long carbon-chain backbones (also known as super carbon-chain compounds) [64]. Ten Symbiodiniaceae-derived compounds have been reported (i.e., **1**–**7** and **10**–**12**) to be closely related structurally to polyketide-like compounds with an amide moiety (see Figure 3).

### 3.4. Challenges Related to the Biotechnological Application of Symbiodiniaceae-Derived Compounds

Studies published from 1962 have described Symbiodiniaceae dinoflagellates as slow-growing microorganisms under culture conditions requiring several weeks to achieve appreciable new colonies [65]. Studies have also reported poor yield of metabolites of interest in culture conditions [66]. These limitations pose challenges in the bioprospecting of Symbiodiniaceae microalgae. Although the yields of several of the isolated compounds are within the expected values (Table 3), significantly high culture volume and time are required to obtain sufficient biomass (an average of 150 L and 47 days; see Table 3). These challenges preclude pharmaceutical applicability which requires large-scale production [64, 67].

Establishing axenic microalgae cultures is a remarkable challenge [68]. Long-term culturing of endosymbionts such as Symbiodiniaceae dinoflagellates can be resource intensive. For instance, a study attempting to achieve a long-term *Cladocopium* (former clade C) culture reported the importance of host conditions for the survival of the Symbiodiniaceae microalgae. However, sustaining a long-term culture was technically unfeasible [69]. Dinoflagellates are sensitive to the hydrodynamic forces in culture media [64, 70–72]. Although immobilized culturing has overcome this limitation [73], it may not be applicable to culturing all Symbiodiniaceae strains, given some strains are obligate endosymbionts. Furthermore, discrepancies between endosymbiotic and free-living states remain incompletely understood [74–76], limiting the respective bioprospecting applications. In addition, the super carbon-chain compounds can also be considered a significant challenge from the chemistry perspective, given the high number of chiral carbons, functional groups, and aliphatic carbons in these metabolites may hinder their structural elucidation. These limitations restrict adequate identification, which in turn limits chemistry-driven studies.

As opposed to primarily establishing axenic cultures, microbial consortia have recently been proven effective in the targeted production of metabolites [77, 78]. In fact, except for studies by Fukatsu et al. [42] and Nakamura et al. [47], the included articles omit describing the axenic condition of the Symbiodiniaceae cultures; only 1 study [42] reported the use of an axenic culture. This implies a putative role of other co-occurring species in the synthesis of the specialized metabolites reported by the included papers.

Among the metabolites reviewed, peridinin (**23**) has been studied the most. This carotenoid pigment forms a molecular complex of significance as a light-harvesting agent for photosynthesis in dinoflagellates (reviewed in detail by Carbonera et al. [79]). Owing to this property, it is currently used as a fluorescent dye in flow cytometry [80]. The study reported producing this compound in a 132 L culture for more than 40 days [44], with a yield of 24.7 mg (Table 3). However, using the immobilized approach and after 28 days of culture, approximately 1 g of peridinin was produced from the *S. voratum* CCAC 0047 strain [81]. This improvement can possibly aid large-scale production and allow exploring other bioactivity potentials of peridinin and other Symbiodiniaceae-derived compounds.

## 4. Conclusions

Although studies on Symbiodiniaceae dinoflagellates are increasing, those on bioprospecting remain considerably low. In addition to a limited number of institutions conducting research on this topic, the challenges in culturing Symbiodiniaceae microalgae, such as slow growth, shear sensitivity, and indeterminate nutrient requirements, contribute to the lack of bioprospecting studies. However, using more adaptable strains (e.g., free-living forms) and culture approaches that prevent hydrodynamic stress (e.g., immobilized growing) could address culturing limitations. These considerations could facilitate more compelling studies by neophyte groups interested in the bioprospecting of dinoflagellates.

This comprehensive literature survey shows that the specialized metabolism of Symbiodiniaceae remains largely unexplored. Future studies should explore new sampling zones, including new hosts (e.g., sponges). Studies are also required to determine genera with free-living stages and in vitro conditions that affect their growth for improved biomass production and better isolation yields.

## Figures and Tables

**Table 1:** Symbiodiniaceae strains and their hosts in the included articles.

Strain code	Strain	Clade	Host^a^	References
S1	*Symbiodinium* sp 2012-7-4S	N/A^b^	*Amphiscolops* sp	[38]
S2	*Symbiodinium* sp HA3-5	A1	Free-living	[39–41]
S3	*Symbiodinium* sp JCUCS-1	B	*Cassiopea xamachana*	[42]
S4	*Symbiodinium* sp JCUSG-1	A	*Sarcophyton glaucum*	[42, 43]
S5	*Symbiodinium* sp OTCL2A	N/A	*Tridacna crocea*	[44]
S6	*Symbiodinium* sp P083-2	A1	*Amphisorus hemprichii*	[43]
S7	*Symbiodinium* sp P-78	D	N/A	[45]
S8	*Symbiodinium* sp PL-TS-1	A3	*Tridacna crocea*	[43]
S9	*Symbiodinium* sp Y-6	A2	*Amphiscolops* sp	[42, 43, 46–48]
S10	*Symbiodinium* sp	N/A	*Pseudopterogorgia elisabethae*	[15, 49, 50]
S11	*Symbiodinium* sp	N/A	*Eunicea fusca*	[50]
S12	*Symbiodinium* sp	N/A	*Amphiscolops* sp	[51–54]

^a^Scientific name of the host from which the *Symbiodiniaceae* strain was isolated. ^b^N/A: not available.

**Table 2:** Symbiodiniaceae-derived metabolites with the reported bioactivity and microalgae strain source.

Compound no.	Metabolite	Bioactivity	Symbiodiniaceae strain	References
**1**	Zooxanthellatoxin A	Vasoconstriction, platelet aggregation	S9	[47, 55, 56]
**2**	Zooxanthellatoxin B	Vasoconstriction	S9	[46, 47]
**3**	Zooxanthellamide A	N/A^b^	S2	[39]
**4**	Zooxanthellamide B	N/A	S2	[40]
**5**	Zooxanthellamide C	Vasoconstriction	S2	[41]
**6**	Zooxanthellamide D	Cytotoxicity	S3	[42]
**7**	Symbiodinolide	N-type Ca^2+^ channel inhibition, COX-2 inhibition (anti-inflammatory)	S12	[53]
**8**	Symbioramide-C16	N/A	S9	[48]
**9**	Zooxanthellactone	Cytotoxicity	S4	[43]
**10**	Symbiospirol A	Protein kinase C inhibition (anti-inflammatory)	S12	[54]
**11**	Symbiospirol B	N/A	S12	[54]
**12**	Symbiospirol C	N/A	S12	[54]
**13**	Symbiodinolactone A	N/A	S1	[38]
**14**	Pseudopterosin A	N/A	S10	[15, 49, 50]
**15**	Pseudopterosin B	N/A	S10	[15, 49, 50]
**16**	Pseudopterosin C	N/A	S10	[15, 49, 50]
**17**	Pseudopterosin D	N/A	S10	[15, 49, 50]
**18**	Fuscol	N/A	S11	[50]
**19**	Symbioimine	Osteoclastogenesis inhibition, COX-2 inhibition (anti-inflammatory)	S12	[51, 52]
**20**	Neosymbioimine	N/A	S12	[52]
**21**	Zooxanthellabetaine A	N/A	S9	[48]
**22**	Zooxanthellamine	N/A	S9	[48]
**23**	Peridinin	Delayed-type hypersensitivity inhibition (anti-inflammatory)	S5	[44]

^a^Assigned compound number. ^b^N/A: not available.

**Table 3:** Summary of the culture conditions and isolation yield data reported by some included articles regarding the biomass production of the compounds isolated from Symbiodiniaceae strains.

Compound no.	Culture time (days)	Culture volume (L)	Wet weight (g)	Compound amount (mg)^b^	Yield × 10^−2^ (%)^c^	References
**1**	30–40	250	164.0	35.8	2.18	[47]
**2**	30–40	250	164.0	19.6	1.20	[47]
**3**	42	198	130.3	4.6	0.35	[39]
**4**	40	132	103.9	5.5	0.53	[40]
**5**	40	132	103.9	21.8	2.10	[41]
**6**	43	140	98.5	2.3	0.23	[42]
**7**	60	78	88.0	9.3	1.06	[53]
**8**	63	160	192.0	2.7	0.14	[48]
**9**	42	156	138.7	1.0	0.07	[43]
**10**	60	145	129.0	117.0	9.07	[54]
**11**	60	145	129.0	9.4	0.73	[54]
**12**	60	145	129.0	3.4	0.26	[54]
**13**	42	100	N/A	0.3	N/A	[38]
**19**	20	80	36.0	5.7	1.58	[51, 52]
**20**	N/A^a^	N/A	112.0	4.2	0.38	[52]
**21**	63	160	192.0	0.8	0.04	[48]
**22**	63	160	192.0	37.6	1.96	[48]
**23**	40	132	82.0	24.7	3.01	[44]

^a^N/A: not available. ^b^Reported amount by authors for the isolated compound. ^c^Calculated/reported yield for the isolated compound.

## Data Availability

The data used to support the findings of this study are provided within this article. However, any required further information can be provided by the corresponding author upon request.

## Supplementary Materials

Supplementary Materials Table S1: PRISMA checklist. Table S2: list of SMILES-annotated compounds retrieved from the included papers. Figure S1: chemical structure of Symbiodiniaceae-derived compounds **1**–**5**. Figure S2: chemical structure of Symbiodiniaceae-derived compounds **6**–**23**.
